# Viscoelastic differences between isolated and live MCF7 cancer cell nuclei resolved with AFM microrheology

**DOI:** 10.1098/rsif.2024.0885

**Published:** 2025-06-18

**Authors:** Ellen Juel Pørtner, Anna Mularski, Tobias William Jarrett, Stine Lauritzen Sønder, Jesper Nylandsted, Adam Cohen Simonsen

**Affiliations:** ^1^ Department of Physics, Chemistry and Pharmacy, University of Southern Denmark, Odense, Denmark; ^2^ Danish Cancer Institute, Membrane Integrity, Danish Cancer Society, Copenhagen, Denmark

**Keywords:** microrheology, cell nucleus, atomic force microscopy, MCF7, viscoelasticity

## Abstract

Cell nuclei are commonly isolated for mechanobiology studies although isolated nuclei may display viscoelastic properties differing from those of live cells. Nuclear mechanics is generally dependent on the time scale of the applied load and cannot accurately be assessed by a simple elasticity parameter. Active microrheology with an atomic force microscope (AFMMR) is a versatile tool for probing nuclear mechanics and we employ the technique for exploring isolated and live-cell nuclei in MCF7 cells, including the significance of actin depolymerization. We successfully validate the method using polyacrylamide hydrogels with correction for cantilever drag in the fluid. The AFMMR results reveal that isolated and live-cell nuclei are equivalent to within a scaling factor, in their frequency-dependent modulus, with isolated nuclei being softer. The loss tangent reveals a transition from solid- to liquid-like behaviour occurring at higher frequency in isolated than in live-cell nuclei. Viscoelastic modelling using the Jeffreys model describes the frequency-dependent modulus of all measured nuclei. Model parameters display sensitivity to nuclei isolation and to actin depolymerization in live cells. Sections of the Jeffreys circuit can potentially be assigned to internal and external nucleus structures, respectively, thereby establishing a minimal mechanistic framework for interpreting microrheology data on cell nuclei.

## 1. Introduction

The mechanical characteristics of cells play important roles in many biological processes, including cell migration [1], metastasis [2] and cell division [3]. The cell nucleus, being the largest and stiffest organelle of the eukaryotic cell [4], is often the dominating factor in determining the whole-cell mechanics. The nucleus is subject to mechanical forces applied by the cytoskeleton and neighbouring cells [5], particularly in cells within tissue exposed to mechanical stress. While it is necessary for cell migration that the nucleus is deformable, it is also vital that the nucleus can withstand and recover from high stresses, which could cause local rupture of the nuclear envelope, leading to DNA damage [6]. Nuclear deformation alone may cause structural changes in chromatin and trigger genetic changes [7]. The nuclear mechanics of cancer cells is of particular importance, as the size and rigidity of the nucleus determine the ability to invade the interstitial space and narrow constrictions such as capillaries [8,9]. Overall, a quantitative characterization of the mechanical properties of the cell nucleus is an important part of characterizing the whole-cell mechanics and for identifying the contribution from nuclear mechanics in specific cell types.

The mechanical response of nuclei, as well as other cellular and sub-cellular structures, can be probed by a wide range of biophysical techniques [10], such as micropipette manipulation [11] and aspiration [12–14], atomic force spectroscopy [15–21], single- or multiple-particle tracking [5,22,23], optical tweezers in isolated [24,25] and live-cell nuclei [26–28] and magnetic nanoparticles in live-cell nuclei [29]. Selecting the appropriate technique largely depends on the specific question being asked including the characteristic length and time scale of the deformation. A point of attention is that the magnitude of the viscoelastic parameters for the *same* cellular system may vary depending on the analytical method used. This was illustrated by Wu *et al*., comparing the mechanical properties of MCF7 cells in eight laboratories measured by six different techniques, showing that the measured mechanical parameters are sensitive to measurement conditions and experimental details including the probe-to-cell contact area, extracellular context, type of deformation, etc. [30].

To measure nuclear mechanics under conditions mimicking the deformation of live cells, tools are required that can impose a deformation of the nucleus at relevant time and length scales and here atomic force microscopy (AFM) is uniquely suited. AFM force versus distance spectroscopy is an established technique for mechanical measurements of cell structures [12,15,17,31], though it is typically performed with indentations at a single approach velocity. This can severely limit the scope of comparison, since biological materials are viscoelastic and display a combination of solid- and liquid-like mechanical behaviour [32]. Thus, mechanical properties acquired at one time scale will typically differ from the mechanical response at other time scales. Therefore, AFM microrheology (AFMMR) is a relevant technique for mechanically characterizing cellular and sub-cellular systems [19,20,33–35]. In AFMMR the indented probe at the end of the flexible cantilever is oscillated at a range of frequencies while in contact with the sample, making it equivalent to macroscopic active rheology, but performed locally on cell microstructures. Using AFMMR, well-known rheological parameters such as the frequency-dependent and complex valued modulus is obtained.

Biophysical measurements of nuclear mechanics are often conducted with isolated nuclei due to practical advantages and decoupling of the nucleus from the cell matrix and the cytoskeleton [11,12,20,24]. However, it has remained rather unclear whether an isolated nucleus displays mechanical features that are equivalent to those of the live-cell nucleus. The isolation process itself involves rupture of the cell and may potentially alter the structural components of the nucleus. Second, the isolated nucleus is decoupled from the crowded cytoplasm and from the endoplasmatic reticulum, which is continuous with the nuclear envelope. Regardless of the source of potential viscoelastic differences, the question is whether isolated nuclei are suitable mechanical models for live-cell nuclei.

Micropipette manipulation experiments conducted on chemically isolated nuclei and nuclei in living cells reported a similar force response [11]. Similarly, micropipette aspiration studies on chemically isolated and nuclei *in vivo* found excellent agreement between their mechanical behaviour [36]. Non-invasive studies agree with these findings: a characterization of nuclear viscosity by multi-particle tracking of chromatin granules in isolated nuclei and those in intact Jurkat cells found no significant differences [23]. It has also been reported, however, that isolated nuclei and nuclei in living cells exhibit different mechanical responses, although the conclusions differ. An AFM study comparing the Young’s modulus of isolated and live valve interstitial cells found the isolated nuclei to be significantly softer than the live-cell nuclei [15]. Conversely, a micropipette aspiration study performed on chemically isolated cartilage cell nuclei measured a significantly higher elasticity and viscosity in comparison with their *in vivo* counterparts [37]. The deviations among these studies may stem from actual differences between the studied cell types, varying methods of nucleus isolation, buffer conditions during measurements, as well as the type of deformation applied to the nuclei; a sharp indenting AFM tip and an aspirating micropipette give rise to different types of deformations, and thus may engage different mechanical components of the nucleus. Therefore, distinct techniques for measuring cellular mechanics can produce varying results on the same system, due to differences in the type of deformation and/or the time and length scales probed.

To fully capture the viscoelastic properties of isolated and live-cell nuclei, a spectrum of relevant time scales must be probed. We, therefore, decided to investigate isolated and live MCF7 cell nuclei with dynamic AFMMR over deformation time scales ranging from milliseconds to seconds. For validation of the method, measurements on reference viscoelastic samples (polyacrylamide (PAA) hydrogel) are included. Dynamic rheology captures the full complex-valued mechanical modulus $E^{*}=E^{\prime }+iE^{\prime \prime }$ as a function of the deformation frequency. The complex modulus is composed of its magnitude $|E^{*}|$, which we for brevity refer to as the *modulus* and the loss tangent, $\tan\;\delta =E^{\prime \prime }/E^{\prime }$ yielding the ratio between the viscous and elastic components of the complex modulus. Using an actin polymerization inhibitor, we examine the contribution from the cytoskeleton to the measured nuclear mechanics in live cells. The data are analysed with viscoelastic circuit models (spring/dashpot type) as previously employed for cell organelles [13,23,24,38]. For the optimal model identified (Jeffreys), we are able to relate variations in circuit component values to changes in the state of the nucleus. Our results suggest that the dominating mechanical changes upon isolation are changes in the intrinsic nuclear response in addition to decoupling of the nucleus from the intracellular matrix.

## 2. Material and methods

### 2.1. Polyacrylamide gel preparation

PAA gels were prepared following the protocol of Tse & Engler [39]. Briefly, acrylamide and bisacrylamide were mixed in DPBS to achieve reported Young’s modulus values as reported in table 10.16.1 of Tse & Engler [39]. Polymerization was initiated by ammonium persulfate and tetramethylethyhlenediamine. To achieve gel adhesion, glass coverslips were amino-silanated.

Three gels were prepared: PAA1 with reported Young’s modulus 0.48 ± 0.16 kPa, 3% acrylamide and 0.06% bisacrylamide; PAA2 with reported Young’s modulus 1.10 ± 0.3 kPa, 3% acrylamide and 0.1 bisacrylamide; and PAA3 with reported Young’s modulus 4.47 ± 1.19 kPa, 5% acrylamide and 0.15 bisacrylamide. All percentages are w/v.

### 2.2. Cell culture

A TNF-sensitive subclone (MCF7S1) of the MCF7 breast cancer cell line is used throughout the study. The MCF7 cell line was provided by Dr David Spriggs, University of Wisconsin (now Massachusetts General Hospital; see also [40]). Cells were cultured in RPMI 1640 Medium, GlutaMAX${,}^{\text{TM}}$ Supplement (ThermoFischer Scientific) with 6% fetal bovine serum (ThermoFischer Scientific) and 0.25% penicillin streptomycin (ThermoFischer Scientific) in an incubator at $37^{\circ }$C with 5% CO${,}_{2}$. The cells were split every 2−3 days with TrypLe${,}^{\text{TM}}$ Express Enzyme (1X) (ThermoFischer Scientific).

Before measurements, the cells were trypsinized and placed on sterilized glass coverslips in the growth medium and allowed to adhere for >12 h. Cells treated with Cytochalasin D (CytoD) (ThermoFischer Scientific, cat. no. PHZ1063) were incubated for 30 min in 2 μM CytoD and rinsed 10 times with media immediately prior to measurement. To ensure that the cells survived this treatment, cell viability assays were performed with the ReadyProbes Cell Viability Imaging Kit (Blue/Red) (ThermoFischer Scientific, cat. no. R37610). The results of these assays are available in electronic supplementary material, figure S3.

### 2.3. Nuclei isolation

Nuclei extraction was performed with the nuclei isolation kit: Nuclei EZ Prep (Sigma-Aldrich, NUC101). All solution and buffers added to the cells during the isolation protocol were ice cold. The cells were treated with a solution of growth medium with 15 μg ml^−1^ digitonin (Sigma-Aldrich, D141) to permeabilize the membrane. The digitonin concentration was optimized to induce minimal cell death (<5%) in MCF7 cells. The cells were left in the digitonin solution on ice on a rocking table for 15 min. Cells were washed in phosphate-buffered saline (Sigma-Aldrich, D8537), and the rest of the protocol follows the technical bulletin from the NUC101 kit. Briefly, hypotonic buffer was added to the cells, which were harvested with a cell scraper and triturated with a P1000 pipette after swelling for 10 min. After isolation, nuclei were stored in Nuclei EZ Storage Buffer at −75${,}^{\circ }$C. Nuclei were isolated from cells between passages 6 and 10, with passage 1 defined as the first plating after the plating following thawing.

For AFM experiments, nuclei were adhered to glass coverslips treated with polyethyleneimine (PEI) (Sigma-Aldrich, P3143). Plasma-cleaned coverslips were submerged in a 0.005% (v/v) solution of PEI in MilliQ for >12 h, then washed with MilliQ. Nuclei were diluted in DPBS without CaCl${,}_{2}$ and MgCl${,}_{2}$ and added to the PEI-coated coverslip and left to adhere for 1 h. Before measurements, the coverslip was flushed with DPBS to remove non-adherent nuclei and other material.

### 2.4. Atomic force microscopy microrheology

AFM experiments were performed with a NanoWizard 4 (JPK, Bruker Nano GmbH), with rectangular cantilevers with a borosilicate glass colloidal probe of radius *R* = 2.5 μm (NanoAndMore, CP-qp-CONT-BSG-A). The cantilevers have a nominal resonance frequency of 30 kHz and a spring constant of 0.1 N m^−1^ in air. The AFM was mounted on a Nikon Ti2-E inverted microscope with a 20× objective (Nikon ELWD S Plan Fluor, NA = 0.6).

The analysis is based on Alcaraz *et al*. [41] and uses the following relation between the measured force F and total indentation $\delta_{T}$ for a parabolic indenter geometry:


(2.1) $$F(t)=\frac{4\sqrt{R_{c}}}{3}\frac{E}{1-\nu^{2}}\delta_{T}(t{)}^{\frac{3}{2}},$$

where E and ν are the Young’s modulus and Poisson’s ratio of the indented material, respectively. The dependence of the force and indentation on time t has been made explicit here. Poisson’s ratio is assumed to be 0.5. $R_{c}$ is the effective radius of the indenter tip curvature.

The indentation depth $\delta_{T}$ is obtained from the vertical position of the tip of the cantilever; this value is based on the cantilever height measured directly from the position of the piezo. Since the cantilever is deflected while it indents, the true tip position takes this deflection into account [42]:


(2.2) $$\delta_{T}=Z-Z_{0}-d,$$

where Z is the cantilever height, $Z_{0}$ the contact point and d the cantilever deflection [32]. For the sake of accuracy, $\delta_{T}$ will be referred to as the vertical tip position.

To include the time dependence of the Young’s modulus, equation (2.1) is considered for low-amplitude oscillations δ around an indentation $\delta_{0}$ by expanding $\delta_{T}^{\frac{3}{2}}=(\delta_{0}+\delta (t){)}^{\frac{3}{2}}$ to its first-order term [41,43], in which case the relaxation modulus E(t) is


(2.3) $$E(t)=\frac{1-\nu^{2}}{2\sqrt{R_{c}\delta_{0}}}\frac{F_{\text{osc}}(t)}{\delta (t)},$$

where $F_{\text{osc}}(t)$ is the force required for the cantilever oscillation and δ(t) is the indentation corresponding to the oscillation. The corresponding frequency-dependent complex modulus, denoted as $E^{*}(\omega )$, may be obtained by a Fourier transformation of equation (2.3) [44]. If $F_{\text{osc}}(\omega )=F_{A}e^{i\phi_{F}}$ and $\delta (\omega )=\delta_{A}e^{i\phi_{\delta }}$ are the Fourier transforms of the force and indentation at angular frequency ω=2πf, with amplitudes $F_{A}$ and $\delta_{A}$ and phases $\phi_{F}$ and $\phi_{\delta }$, then


(2.4)E∗(ω)=E′(ω)+iE″(ω)(2.5)=1−ν22Rcδ0FAδAexp⁡[iΔϕ],

where $\Delta_{\phi }(\omega )=\phi_{F}-\phi_{\delta }$ is the phase difference between the force and the deformation.

The total force on the cantilever is a sum of the hydrodynamic drag force caused by viscous friction with the surrounding medium and the force response of the sample. Corrections for the hydrodynamic drag acting on the oscillating cantilever are based on Alcaraz *et al*. [45]; details and results are found in electronic supplementary material, figure S1A. For this correction, the heights of nuclei are required; these were estimated with AFM force curves as described in electronic supplementary material, figure S1B.

After correction for the hydrodynamic drag, the expression for the complex modulus $E^{*}(\omega )$ is


(2.6) $$E^{*}(\omega )=\frac{1-\nu^{2}}{2\sqrt{R\delta_{0}}}\frac{F_{A}}{\delta_{A}}\exp\left[i\Delta_{\phi }-i\frac{\pi }{2}\omega b(h)\right],$$

where b(h) is the drag coefficient, evaluated at the height of the nucleus h.

**Figure 1.**
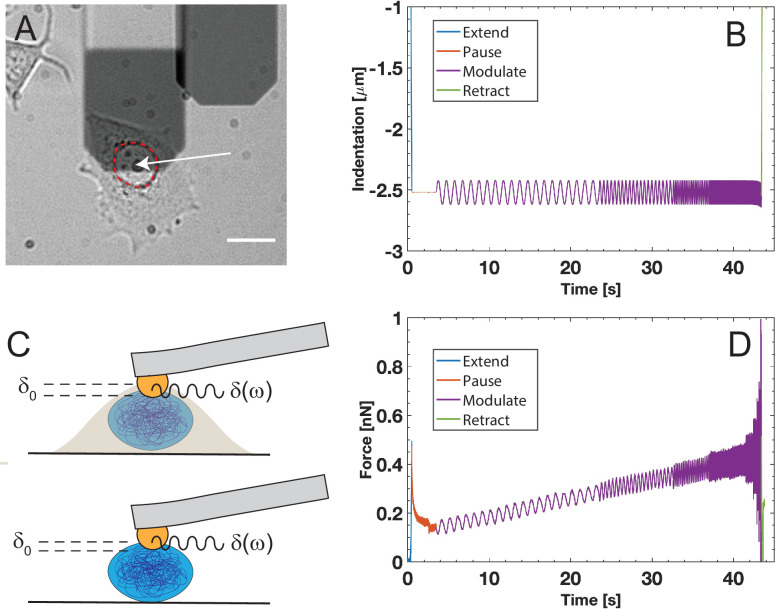
Principle of dynamic AFM microrheology: (A) cantilever positioned above a live-cell nucleus as highlighted by the red dashed line. The colloidal probe is located at the end of the cantilever as indicated by the arrow (scale bar: 20 μm). The probe indents the nucleus until a pre-defined force setpoint is reached. The corresponding indentation is $\delta_{0}$. The cantilever is held at fixed height for 3 s to allow for relaxation of the material, followed by a modulation of the indentation (B). During modulation, the cantilever indentation oscillates in feedback at increasing frequencies ω. The position of the cantilever relative to $\delta_{0}$ is defined as δ(ω) (C). Typical recording on a live-cell nucleus with indentation (B) and force (D).

Nuclei were indented with a setpoint value of 0.5 and 1 nN with a speed of 15 μ ms^−1^, followed by a 3 s pause at constant height (figure 1B). The cantilever was set to oscillate for 20 periods with an amplitude of 100 nm (figure 1B). For each setpoint value, 10 modulation frequencies in the range [1,220] Hz were applied. The sampling rate was varied for each frequency, such that at least 100 points per period were recorded.

All experiments were conducted at $37^{\circ }$C. The cells were measured in their growth medium, and the isolated nuclei in PBS without CaCl${,}_{2}$ and MgCl${,}_{2}$. Cells were chosen for measurement such that they were not visibly in contact with other cells; see figure 1A. Each experiment lasted no longer than 1 h to ensure cell viability in the absence of CO${,}_{2}$ control.

### 2.5. Confocal microscopy

Confocal microscopy was performed with a Nikon AX microscope using a 60× objective (Nikon CFI Plan Apochromat λD, NA = 1.42). Fluorescence excitation was with a LU-NV laser unit. Images were captured by a GaAsP detector with a 2048 × 2048 pixel resolution, emitting lasers in sequence for minimal crosstalk between channels. Isolated nuclei and live cells were stained with Hoechst 33342 (NucBlue${,}^{\text{TM}}$ Live ReadyProbes${,}^{\text{TM}}$ ThermoFischer Scientific) with 2 drops ml^−1^ for 20 min in PBS at room temperature and excited using a 405 nm laser line.

For the rhodamine phalloidin staining, cell medium was removed and the cells were covered with 4% paraformaldehyde for 20 min at room temperature. After rinsing with PBS, cells were treated with 0.1% (v/v) Triton X-100 in PBS for 10 min. Following another rinse with PBS, cells were incubated in 1% (w/v) bovine serum albumin (BSA) in PBS for 1 h. The BSA solution was removed, and the cells were incubated in a 165 nM rhodamine phalloidin (Invitrogen) solution for 30 min, then 2 drops ml^−1^ Hoechst 33342 for 10 min. Rhodamine phalloidin excitation was achieved using a 561 nm laser line.

Images were processed in Nikon imaging software NIS-Elements Advanced Research v. 5.30.02. All frames were denoised with the Advanced Denoising command with the ‘Original’ algorithm and denoising power 1.0 for all channels.

### 2.6. Viscoelastic modelling

The viscoelastic circuit models are described in detail in electronic supplementary material, figure S2. Fitting of the viscoelastic models to the AFMMR rheology data was performed in Matlab using the ‘fmincon’ optimization method for multi-parameter fitting. Fitting was restricted to the modulus parameter $|E^{*}|$. Statistical tests comparing the fit parameters for the Jeffreys model were performed using the Welch and Brown–Forsythe ANOVA tests for three or more sets of unpaired measurements.

## 3. Results and discussion

### 3.1. Validation of AFMMR with a reference gel

Before proceeding to characterize isolated and live-cell nuclei, we decided to validate the methodology to ensure that AFMMR is capable of detecting viscoelastic differences in reference samples with material properties comparable to nuclei. For this, we used a series of hydrogels (PAA) with known values of the Young’s modulus. Hydrogels are often used as substrates for cell cultures, where the viscoelastic properties of the hydrogel have been shown to influence cell survival, morphology, differentiation and protein expression [46–49]. Thus, although hydrogels do not fully mimic the viscoelastic response of cell nuclei, they can be made with comparable moduli and are established rheological samples [50–53], which make them suitable as reference for validation purpose.

**Figure 2.**
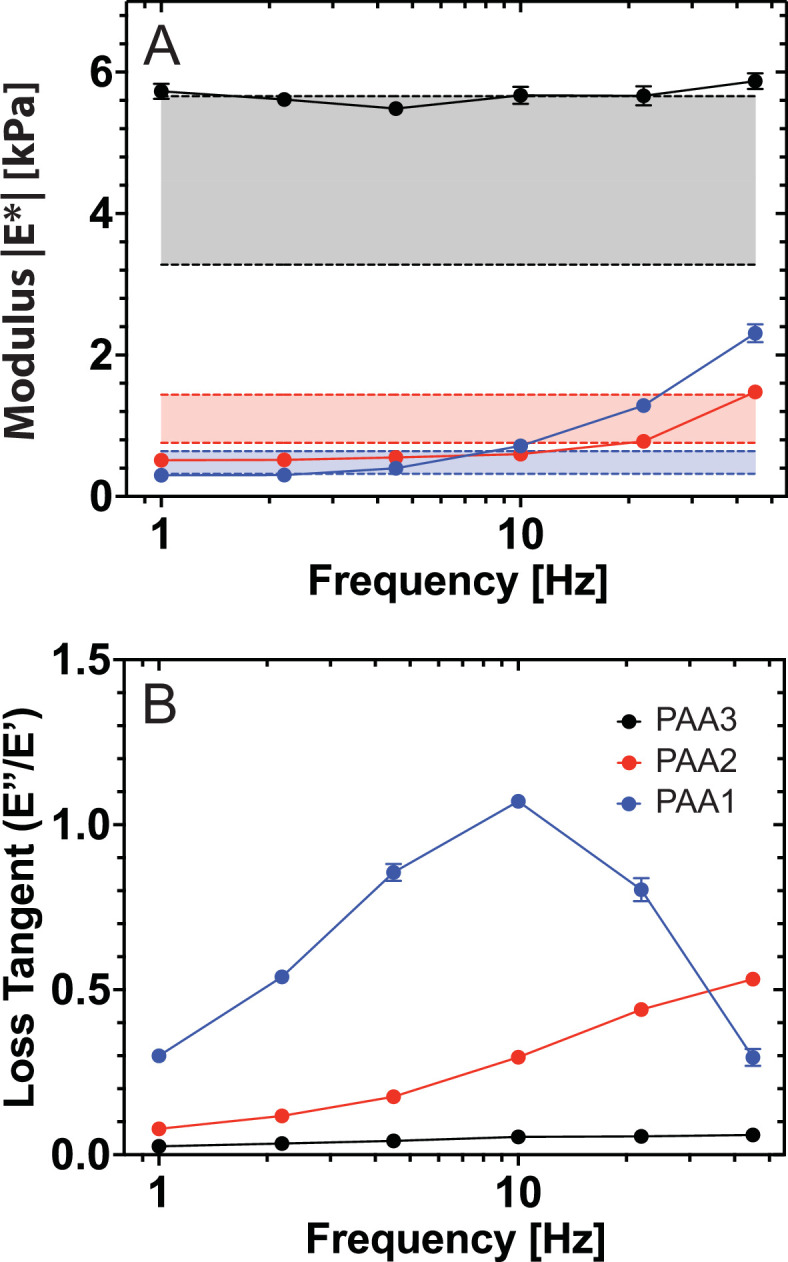
AFM microrheology of polyacrylamide hydrogels for validation. The modulus $|E^{*}|$ (A) and the loss tangent (B) of the hydrogels are shown as mean ± standard error of the mean. Shaded bands show published frequency-independent AFM indentation results [39] (mean ± standard deviation). The loss tangent resolves a changing ratio between the viscous and elastic response of the hydrogels and correlates with a decreasing ratio $\frac{\left[A\right]}{\left[BA\right]}$ of acrylamide and bisacrylamide concentrations in samples PAA1 to PAA3.

We used three PAA hydrogels with reported Young’s moduli in the same range as previously reported modulus values for cell nuclei [20]. The reported Young’s modulus *G* for each of the gels is [39]: *G*(PAA1) = 0.48 ± 0.16 kPa, *G*(PAA2) = 1.10 ± 0.3 kPa, *G*(PAA3) = 4.47 ± 1.19 kPa. The gels were >1 mm thick and fixed to glass slides by silanization. They were measured in PBS buffer with the same microsphere AFM probes used on cell nuclei. The range of Young’s moduli reported by Tse & Engler [39] (no frequency dependence) is shown as bands in figure 2A for comparison.

**Figure 3.**
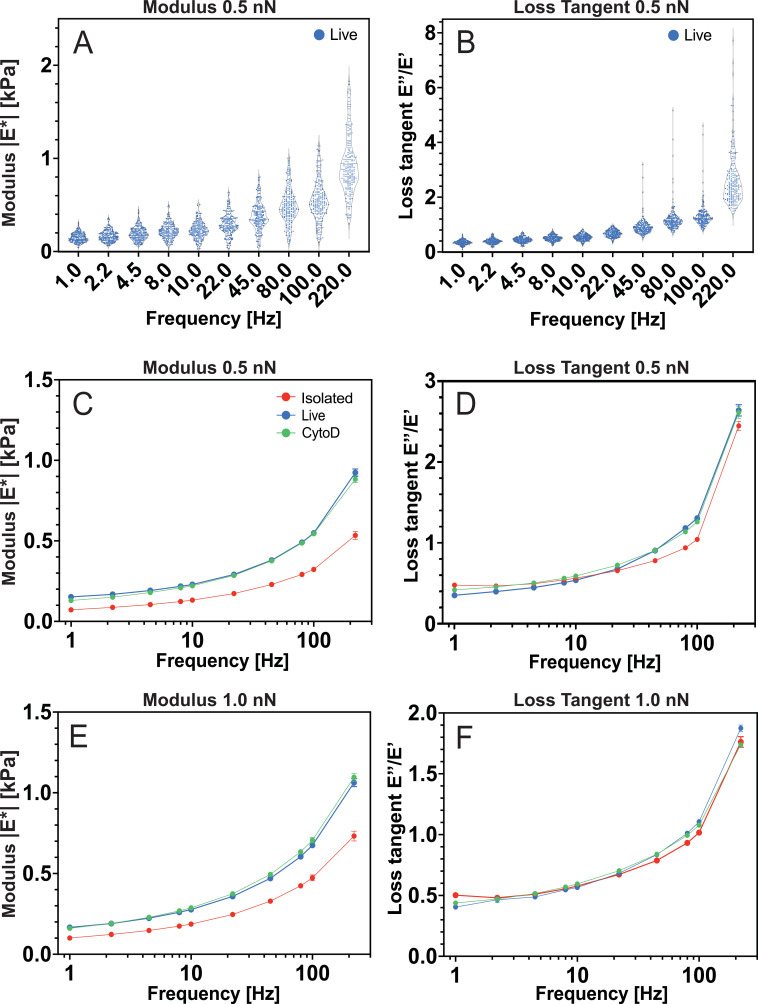
Active microrheology data on MCF7 cell nuclei as probed by AFMMR with indentation forces of 0.5 nN and 1 nN. Shown are the modulus $|E^{*}|$ (A,C,E) and loss tangent (B,D,F) of isolated nuclei (n=50), live-cell nuclei (n=50) and nuclei in cells treated with 2 μM CytoD (n=50). Data in (A,B) show the full dataset for live nuclei at 0.5 nN while (C–F) show mean values ± standard error of the mean.

At low frequencies (i.e. long time scales), the hydrogels behave as elastic solids, as indicated by the plateau of the modulus (figure 2A) and a loss tangent well below 1 (figure 2B). This behaviour is most pronounced for the stiffest gel (PAA3), as also found with dynamic light scattering [52]. As the stiffer PAA gels have a higher density of cross-links this increases the elastic strength. At 1 Hz, the soft gels PAA1 and PAA2 have a measured modulus of 0.3 and 0.5 kPa, respectively, which are comparable to the values for nuclei at the same frequency (figure 3A).

A clear variation in the viscoelastic response when increasing the concentration of cross-linking bisacrylamide (PAA1 to PAA2) is shown in figure 2B. The loss tangent of the PAA1 gel displays a peak around 10 Hz as also reported by Abidine *et al.* [50]. The peak indicates a possible transition to the glassy state, above which the chains of the polymer gel do not have time to rearrange and dissipate energy [54].

As expected, the loss tangents in figure 2B show an overall decrease with increasing concentration of bisacrylamide, except for PAA1 above 30 Hz. This can be explained by a higher density of cross-linkers that shorten the relaxation times when the material shifts to a more solid-like behaviour and is in line with previous reports that the loss tangent of PAA hydrogels depends inversely on the concentration of the bisacrylamide cross-linker [55].

Overall, the AFMMR measurements on PAA hydrogels demonstrate agreement with previously reported modulus values while revealing frequency variations that are hidden in simple indentation measurements. It also demonstrates that variations in the viscoelasticity of materials comparable to nuclei are clearly resolvable with AFMMR.

### 3.2. Morphology of isolated and live-cell nuclei

**Figure 4.**
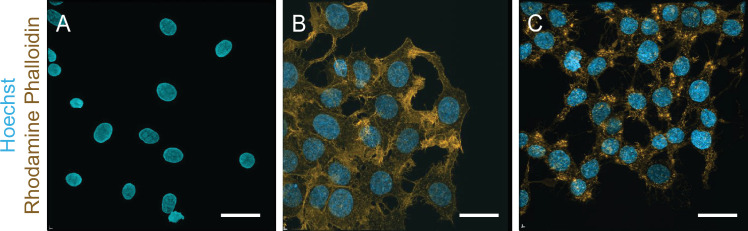
Maximum intensity projection of the z-stack of isolated nuclei adhered to glass coverslips with PEI (A), untreated MCF7 cells (B) and cells incubated in 2 μM CytoD. Both isolated nuclei and cells were stained with the nucleic acid stain Hoechst 33342 and with the F-actin stain rhodamine phalloidin to monitor actin filament disruption by CytoD. Scale bar, 25 μm.

Previous AFM results indicate that the cytoskeleton has an impact on the nuclear mechanical response where measured nuclear moduli increase with increased cytoskeletal tension [15]. To probe this effect in our comparison of isolated nuclei and live-cell nuclei, we include studies of live-cell nuclei treated with the inhibitor CytoD to compromise actin polymerization. Cells were treated with 2 μM CytoD for 30 min and to confirm the effect of CytoD treatment on F-actin, confocal imaging was performed (maximum intensity projection). Figure 4A first shows isolated nuclei absent of actin fibres. Second, the live, untreated cells in figure 4B display an intact cytoskeleton and lastly the CytoD-treated cells in figure 4C display severe actin disruption.

**Figure 5.**
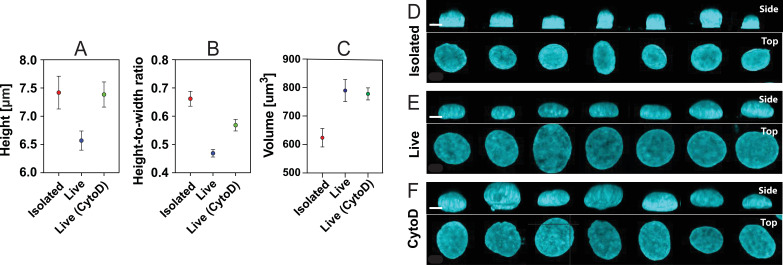
Morphological comparison of MCF7 cell nuclei based on confocal microscopy. Height, height-to-width ratio and volume (A–C) of nuclei calculated based on fluorescence z-stack three-dimensional projections (*n* = 59 isolated, *n* = 59 live, *n* = 82 CytoD). Results are shown as mean ± standard error of the mean. Side view and top view of isolated nuclei (D), live-cell nuclei (E) and nuclei in cells treated with 2 μM CytoD (F), obtained from fluorescence microscopy z-stacks. All nuclei are stained with Hoechst 33342. Scale bars, (D–F) 5 μm.

Next, as a foundation for the rheological measurements by AFMMR, we measured the height and aspect ratio of the nuclei with confocal microscopy. From the sideview projections of z-stacks in figure 5D–F, isolated nuclei are on average 0.85 μm taller than the untreated live-cell nuclei, but only 0.03 μm taller than the nuclei in cells treated with CytoD (figure 5A). The isolated nuclei, but not the live-cell nuclei, are flattened at the bottom due to their adhesion to the PEI-coated coverslip.

The nuclear aspect ratio (height/width) was determined from the confocal three-dimensional projections and is shown in figure 5B. The aspect ratios of the live-cell nuclei have average values of 0.47 and 0.57 for the untreated cells and the CytoD cells, respectively. In comparison, the isolated nuclei have a larger aspect ratio of 0.66. Moreover, the volume of isolated nuclei was found to decrease in comparison with live-cell nuclei with and without CytoD (figure 4C). Shrinking and rounding of nuclei upon isolation have been associated with a loss of compressive pressure from the cytoskeleton [56] and nuclear volume has been found to be regulated by osmotic effects [57]. It is supported here by the increase in the aspect ratio of live-cell nuclei treated with CytoD in comparison with untreated cells and a volume decrease upon isolation.

In addition to the heights obtained by confocal microscopy, the absolute height of the nuclei was also obtained by AFM force versus distance curves combining a measurement on the nuclei with one on the glass surface to identify the background height. Measuring the height of the nuclei is important for two reasons: (i) the absolute height of the cantilever above the substrate is required for the correction of hydrodynamic drag and (ii) identifying potential differences in the geometry of isolated versus live-cell nuclei, which could influence the measured mechanical properties. The details underlying the correction for hydrodynamic drag at a specific height are described in detail in the electronic supplementary material.

### 3.3. Microrheology of isolated and live-cell nuclei

In the AFMMR measurements, isolated and live-cell nuclei were probed at frequencies between 1 and 220 Hz, for setpoint forces of 0.5 and 1 nN, respectively. The nucleus is first indented by the spherical probe until the force setpoint is reached. After a 3 s wait, in which the probe is held at a fixed height, the cantilever is oscillated with an amplitude of 100 nm for 20 periods while in feedback. The oscillation amplitude must be small enough to fulfil the criteria of the Hertz model, yet large enough to result in forces detectable by the AFM at all frequencies of the experiment, in particular at low frequencies. The oscillating force required to maintain the oscillating indentation is detected and used to compute the viscoelastic parameters. Examples of typical AFMMR measurements are shown in figure 1B,D. The microrheology data consist of the modulus $|E^{*}|$ and the loss tangent $\tan\;\delta =\frac{E^{\prime \prime }}{E^{\prime }}$, indicating whether the material is predominantly viscous (tan⁡δ>1) or elastic (tan⁡δ<1) at a given oscillation frequency.

A summary of AFMMR data on all samples is shown in figure 3. A general observation is that the modulus of all nuclei increases with frequency (figure 3A,C,E), as is typical for a viscoelastic material. Also, the modulus is overall highest at the highest indentation force for all frequencies and all nuclei. At the lowest modulation frequency of 1 Hz, which is most comparable to the approach speed of a typical AFM indentation measurement without modulation, the isolated nuclei show a mean modulus of |E∗|=0.07 kPa and |E∗|=0.1 kPa at 0.5 and 1 nN, respectively. The live-cell nuclei are almost twice as stiff, with corresponding values of |E∗|=0.15 kPa and |E∗|=0.17 kPa. The mean modulus of the nuclei in cells treated with CytoD is slightly lower than in untreated cells, with |E∗|=0.13 kPa and |E∗|=0.16 kPa. A similar difference between the modulus of the samples persists at frequencies above 1 Hz. The decreased modulus after isolation may be attributed to a change of chromatin compaction and/or compositional changes of the nucleoplasm as indicated by the change in nuclear morphology in figure 5A–C.

While the modulus increases with the setpoint force for all nuclei, the effect of the setpoint force is less pronounced when considering the loss tangent. For the live-cell nuclei in figure 3D,F, the loss tangent is relatively independent of force for frequencies <45 Hz, while at higher frequencies, the live-cell nuclei are more viscous at the lowest indentation force. An increase in the loss tangent for decreasing indentation force indicates that the outer regions are more viscous. The greater variation of the loss tangent of the live-cell nuclei as a function of force, as compared with the isolated nuclei, suggests that the live-cell nuclei are more heterogeneous in composition than their isolated counterparts. The loss tangents in figure 3D,F show that the isolated and CytoD-treated cell nuclei display an overall more elastic mechanical response at 0.5 nN force while the difference in loss tangent between nuclei is absent at the higher force of 1 nN.

**Figure 6.**
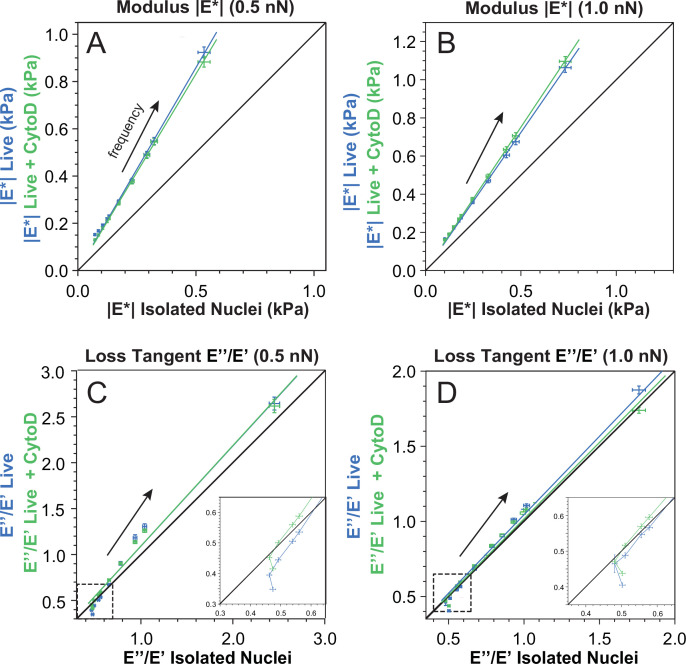
Correlations of the modulus and the loss tangent between isolated and live MCF7 cell nuclei. Live-cell data are shown on the *y*-axis while data for isolated nuclei are on the *x*-axis (A–D). Error bars are ± standard error of the mean. Solid lines are linear fits and arrows indicate the direction of increasing frequency and the black lines show equality (y=x). Data above the black line indicate that live-cell nuclei are stiffer (A,B) or have a higher loss tangent value (C,D) than isolated nuclei for a given frequency and setpoint force.

A comparison of the mechanical properties of the isolated nuclei and nuclei in live cells and live cells treated with 2 μM CytoD, respectively, is shown in figure 6, in which the mean modulus and mean loss tangent of the live-cell nuclei are plotted as a function of the corresponding values for the isolated nuclei.

The modulus $|E^{*}|$ of the live-cell nuclei, with and without CytoD, is consistently larger than that of the isolated nuclei (figure 6A,B) for all frequencies. The slope of the linear fit of the modulus in figure 6A,B for the untreated live-cell nuclei is 1.72±0.04 at 0.5 nN and 1.45±0.2 at 1 nN; for the CytoD-treated cells, the corresponding slopes are 1.66±0.02 and 1.50±0.01. Furthermore, with the exception of the modulus measured at $F_{sp}=0.5$ nN at 1 Hz, there is no significant difference in the size of the modulus of the nuclei in cells treated and not treated with CytoD, as confirmed by a Student’s *t*‐test. Thus, the greater modulus of the live-cell nuclei as compared with the isolated nuclei cannot be assigned to the effect of the actin cytoskeleton. A possible origin is that modulus changes are attributed to differences in chromatin compaction between the isolated and the live-cell nuclei.

To further examine this hypothesis, we attempted to induce chromatin compaction in isolated nuclei by introducing divalent cations to the buffer [12]. Comparing AFMMR on isolated nuclei in a buffer with 2 mM Ca${,}^{2+}$ and 2 mM Mg${,}^{2+}$ to nuclei without (see electronic supplementary material, figure S4), confirms that ion-induced chromatin compaction leads to an increased modulus at all probed frequencies. The loss tangent of the isolated nuclei with Ca${,}^{2+}$ is decreased as compared with isolated nuclei without, for all frequencies. This is consistent with an increasing chromatin compaction and a more solid/elastic response of the nucleus. However, as shown below, the loss tangent of the live-cell nuclei is not decreased relative to the isolated nuclei, although their modulus is higher. This suggests that the mechanical effect of chromatin compactions in live cells can only partially be recovered by introducing divalent cations to isolated nuclei.

The frequency-dependent modulus for isolated and live-cell nuclei essentially differ only by a multiplicative factor. In contrast, the loss tangent displays more subtle frequency-dependent variations highlighting its importance as a descriptive parameter in mechanical analysis. This is illustrated in figure 6C,D showing a transition with respect to frequency: at low frequencies (≤10 Hz) the loss tangent of isolated nuclei is smaller than for the live-cell nuclei. But at higher frequencies, the sequence is reversed. This is indicated by the separation of the data points from the y=x line in figure 6C,D (inset). Moreover, for cells without CytoD, this transition occurs between 10 and 22 Hz for both setpoint forces whereas when cells are treated with CytoD, the transition occurs at lower frequencies. Specifically, the transition occurs at 2.2 Hz at 0.5 nN and between 2.2 Hz and 4.5 Hz at 1 nN. Thus, actin removal by CytoD makes the live-cell nuclei respond more like the isolated nuclei at low frequencies (<10 Hz). A *t*‐test confirms a statistically significant difference between the nuclei in treated and untreated cells in the low-frequency regime. At higher frequencies (>10 Hz), the loss tangent of the live-cell nuclei is larger than that of the isolated nuclei (figure 6C,D), and thus they respond comparatively more like a liquid. A *t*‐test comparing the loss tangents of nuclei with and without CytoD found no significant difference, indicating that the loss tangent is not influenced by the cytoskeleton above 10 Hz.

Overall, the relative variations in the loss tangent between isolated and live-cell nuclei are small, although significant, in comparison with the larger variations in the modulus parameter $|E^{*}|$. The actin cytoskeleton mainly plays a role in low-frequency deformations. The observed mechanical changes appear to be driven by alterations of mechanically significant nuclear components during the isolation process. However, these changes can only partly be reversed by simple ionic compaction of the isolated nuclei. We stress that when nuclei are isolated they not only lose the interactions with actin but also with cytoskeletal filaments (microtubules, intermediate filaments); the LINC complex [58] is disrupted and the continuity between the nuclear membrane and the endoplasmic reticulum is broken. The changes in nuclear morphology and volume support significant structural changes underlying the mechanical changes.

The similarity in the shape of the frequency-dependent mechanical response between isolated and live-cell nuclei is striking. Both the loss tangents and complex moduli of the isolated and live-cell nuclei show similar trends, which may indicate that the mechanical response stems mainly from the equivalent physical elements within the nucleus. We hypothesize that the differences in modulus upon isolation are more related to modified values of the physical components rather than a reconfiguration of the mechanical circuitry of the nucleus. To further test this hypothesis we have pursued viscoelastic modelling of the AFMMR data.

### 3.4. Viscoelastic modelling

**Figure 7.**
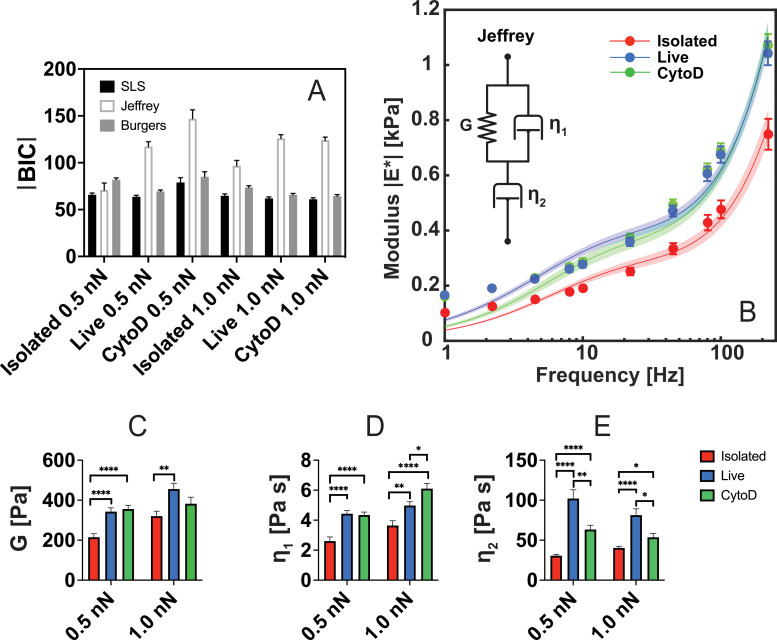
Viscoelastic circuit models of AFMMR data for cell nuclei. Three models (SLS, Jeffreys and Burgers) were fitted to the modulus |E*| of nuclei (*n* = 50). The Bayesian information criterion (BIC) is used for model selection (A). Jeffreys model has the overall highest |BIC| values, and is chosen for fitting. Fitting of Jeffreys model to the mean modulus |E*| for nuclei at 1.0 nN, with shaded regions indicating the standard error mean (B). Jeffreys model contains three components, which are shown in bar plots with indicators of statistical significance: The spring G (C), the dashpot $\eta_{1}$ in parallel with the spring (D), and a second viscous element $\eta_{2}$ (E). Error bars in (C)*–(*E) indicate the standard error of the mean. No marker indicates non-significance (*p* > 0.05) while asterisks indicate the significance level: *(*p* < 0.05), **(*p* < 0.01), ***(*p* < 0.001), ****(*p* < 0.0001).

To interpret the frequency-dependent mechanical behaviour of isolated and live-cell nuclei, we examined three viscoelastic circuit models with respect to fitting the microrheology data. Spring–dashpot circuit models are phenomenological models commonly employed to model the properties of viscoelastic materials and have been used successfully to describe the deformation of nuclei [24,38,59–61]. The models are constructed by combining elastic (spring) and viscous (dashpot) components in series or in parallel. Three common spring–dashpot models have been tested with respect to fitting the microrheological data as shown in figure 7: the standard linear solid (SLS) model, the Jeffreys model and the Burgers model. The two first contain three circuit components while the Burgers model contains four components. The model equations are outlined in detail in the electronic supplementary material. Here, we have chosen viscoelastic models over power law fitting, since the components of viscoelastic models may potentially be associated with structures of the cellular system.

Since the magnitude of the modulus, rather than the loss tangent, displays the largest variations between isolated and live-cell nuclei, we have fitted the models to the frequency-dependent modulus. The models were fitted to data averaged over replicates.

When doing model selection among models with different numbers of fitting parameters, the possibility of overfitting must be considered. We, therefore, quantitatively compare the models using the Bayesian information criterion (BIC), which penalizes models with a larger number of fitting parameters. In general, models with a higher value of |BIC| are preferred. As seen in figure 7A, the Jeffreys model appears as the overall most optimal model for fitting to the modulus data, although for isolated nuclei at 0.5 nN, all models are equivalent according to the BIC. For the purpose of comparison, we seek a common model for all samples and conditions, and therefore continue the analysis using the fitted parameters as described by Jeffreys model. It is also referred to as a three-element liquid and contains a dashpot in series with a Kelvin–Voight (KV) segment consisting of parallel dashpot and spring components (figure 7B). Jeffreys model successfully captures most of the frequency spectrum of the measured modulus values although slight deviations are found at the lowest frequencies (<4 Hz). The three parameter values obtained by fitting to the Jeffreys model are compared in figure 7C–E including statistical tests of significant differences among the sample types, as denoted by the asterisks.

Within the KV segment, the values of both the elastic element G and of the viscous element $\eta_{1}$ are significantly lower for the isolated nuclei than for the live-cell nuclei both with and without CytoD, as shown in figure 7C,D. However, for the live cells, there is no significant difference between cell nuclei with and without CytoD as indicated by the elements G and $\eta_{1}$. Thus, while isolated and live-cell nuclei are clearly distinguished by the KV segment parameters, the effect of actin depolymerization is not. However, the dashpot component $\eta_{2}$ outside the KV element has a statistically significant sensitivity to both the treatment with actin and to isolation of the nuclei such that all three cases can be distinguished by the variation of $\eta_{2}$, as shown in figure 7E. The untreated live-cell nuclei have the highest value of $\eta_{2}$ that is decreased upon actin depolymerization with CytoD in live cells and further reduced for the isolated nuclei. Overall, the viscous modelling results are compatible with the interpretation that the KV segment mainly describes the intrinsic mechanics of the nucleus. This part of the model circuit is relatively unaffected by actin depolymerization in live cells, but displays decreasing component values upon isolation of the nuclei. In contrast, the dashpot component $\eta_{2}$ is sensitive to both actin depolymerization and isolation. This indicates that $\eta_{2}$ is sensitive to the viscous mechanical response of cell structures outside the nucleus. These external structures comprise both actin and other mechanical components of the cell. Removal of actin lowers the value of $\eta_{2}$ while isolation removes all remaining mechanical structures outside the nucleus and further reduces $\eta_{2}$. The interpretation is further substantiated by the fact that Jeffreys model is the minimal viscoelastic circuit capable of replicating the frequency-dependent modulus and that it is preferred over the four-component Burgers circuit according to the Bayesian information criterion. The identification of Jeffreys circuit as a workable minimal viscoelastic model for both isolated and live-cell nuclei may be a helpful reference frame in microrheology measurements on cell nuclei and for interpreting variations between distinct cell types including varying environmental conditions.

## 4. Conclusion

The frequency-dependent viscoelasticity of cell nuclei is potentially altered when nuclei are isolated from the cell. Using dynamic AFMMR over a wide frequency range, we have examined viscoelastic differences between isolated and live MCF7 cell nuclei including the effect of compromising actin polymerization in live cells. Initial validation using a PAA gel yielded excellent agreement with reference mechanical moduli and showed sensitivity of the rheological response to the time scale of deformation.

Confocal fluorescence imaging revealed that isolated nuclei have a larger height-to-width aspect ratio than the corresponding nuclei in live cells, indicating chromatin decompaction upon isolation. The difference correlates with the presence of the actin cytoskeleton in live cells. AFMMR measurements, corrected for hydrodynamic drag and validated with test PAA samples, reveal that isolated and live-cell nuclei are equivalent in their frequency-dependent modulus, within a constant factor, suggesting that the mechanically dominant structures of the nuclei are preserved after isolation. However, distinct differences are detected by the loss tangent between the three systems studied. In particular, the frequency at which the nucleus transitions from solid- to liquid-like response is higher in isolated nuclei than in live cells. This highlights the importance of the loss tangent as a viscoelastic descriptor, as it reports differences in mechanical behaviour that may not be detected by the overall modulus alone.

We find that the three-parameter Jeffreys model can be used to describe the frequency-dependent modulus of the nucleus from 1 to 200 Hz and that it is superior to the four-component Burgers model according to the Bayesian information criterion. Variations in the component values suggest that distinct parts of the Jeffreys circuit can potentially be assigned to the intrinsic nuclear response and to structures outside the nucleus, respectively.

## Data Availability

Data for figures are available via Zenodo [62]. Electronic supplementary material is available online [63].
